# Successful treatment of severe *Pneumocystis* pneumonia in an immunosuppressed patient using caspofungin combined with clindamycin: a case report and literature review

**DOI:** 10.1186/s12890-016-0307-0

**Published:** 2016-11-11

**Authors:** Hongjuan Li, Haoming Huang, Hangyong He

**Affiliations:** 1 Department of Emergency, Guangdong Hospital of Traditional Chinese Medicine, Guangzhou, Guangdong 510105 China; 2 Department of Emergency, Guangzhou University of Traditional Chinese Medicine First Affiliated Hospital, Guangzhou, Guangdong 510405 China; 3 Department of Respiratory and Critical Care Medicine, Beijing Chao-Yang Hospital, Capital Medical University, Beijing, 100020 China

**Keywords:** *Pneumocystis* pneumonia, Caspofungin, Clindamycin

## Abstract

**Background:**

*Pneumocystis jirovecii* is responsible for *Pneumocystis* pneumonia (PCP), which occurs almost exclusively in immunocompromised individuals. Trimethoprim-sulfamethoxazole (TMP-SMZ) is regarded as the first-line treatment and prophylaxis for *P. jirovecii* infection, but the frequency of adverse reactions and newly emerged antibiotic resistance limit its use.

**Case presentation:**

Ulcerations and hemorrhages involving the tongue were noted secondary to TMP-SMZ desensitization against PCP in a 46-year-old male who had previously been diagnosed with IgA nephropathy and sustained prolonged corticosteroid therapy. There was an urgent need for an alternative regimen due to the severe response to TMP-SMZ. The patient was successfully treated with a combination therapy of caspofungin and clindamycin.

**Conclusion:**

Caspofungin combined with clindamycin is an optional treatment for PCP when treatment with TMP-SMZ fails or in patients who cannot tolerate TMP-SMZ.

## Background

*Pneumocystis* pneumonia (PCP) is an opportunistic infection caused by *Pneumocystis jirovecii*, which mainly occurs when cellular immunity is depressed because of AIDS, malignancies, prolonged corticosteroid therapy, or organ transplantation. Recent research has indicated that underlying renal dysfunction and chronic renal pathology are risk factors for PCP in patients with IgA nephropathy [1]. The PCP mortality rate is high among patients with delayed diagnosis and treatment, and death is due to severe respiratory failure [2, 3].

The first-line medication of treatment and prophylaxis for *P. jirovecii* infection is trimethoprim-sulfamethoxazole (TMP-SMZ) [4]; however, use of TMP-SMZ could be problematic in patients with adverse reactions and drug resistance. Caspofungin-based therapy has been shown to be effective against *Pneumocystis* in animal models of PCP [5–7]; however, the clinical experience with caspofungin in human PCP are limited and controversial. Herein, we report a case involving salvage therapy with caspofungin and clindamycin in the successful management of an immunosuppressed PCP patient who was allergic to TMP-SMZ.

## Case presentation

A 46-year-old male had been diagnosed with IgA nephropathy based on renal biopsy 3 months before admission. A concurrent diagnosis of chronic kidney dysfunction was established. He was treated with cyclophosphamide and high-dose methylprednisolone, followed by methylprednisolone (40 mg orally per day) for maintenance. He also had hypertension, diabetes mellitus, gout, and leukoderma. He was shown to be allergic to TMP-SMZ when treated for a respiratory infection some years before. The allergic reaction manifested as ulcerations involving the tongue and genitalia, which resolved gradually with discontinuation of TMP-SMZ.

**Fig. 1:**
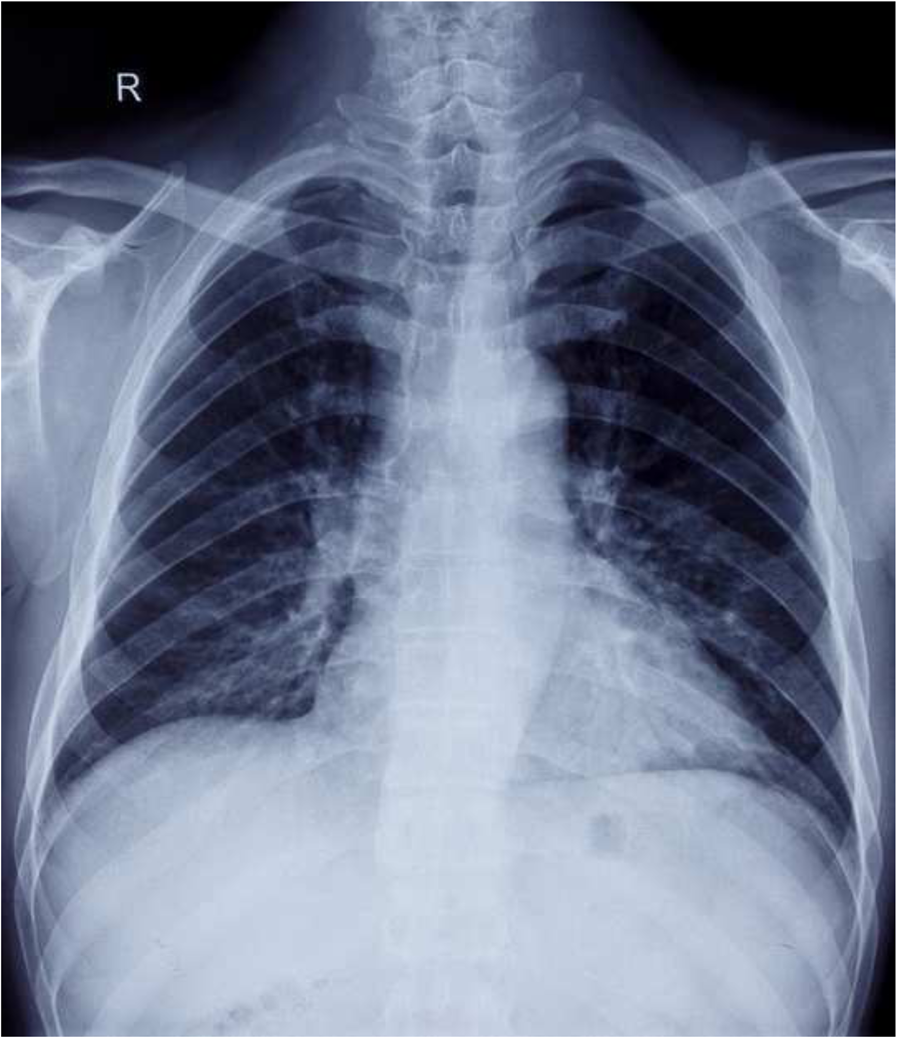
Posteroanterior chest X-ray image one week before the patient’s transfer to the respiratory intensive care unit (RICU). No significant finding was observed on the X-ray image at this date (December 2015)

**Fig. 2:**
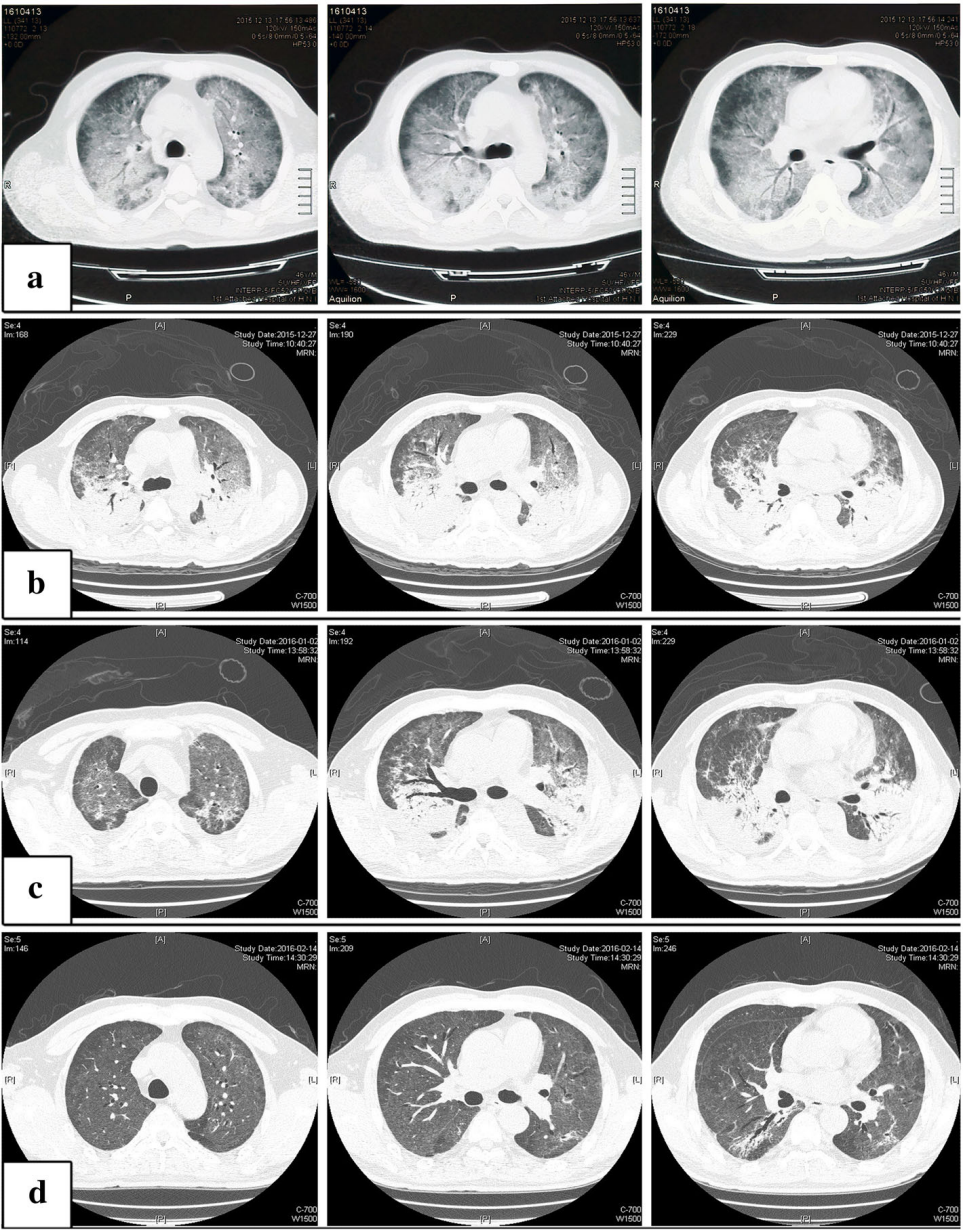
High resolution CT scans of the chest at the levels of aortic arch, root of ascending aorta and pulmonary arteries from left to right, performed from top to down on days 1 (**a**: December 2015), 14 (**b**: December 2015), 20 (**c**: January 2016) and 90 (**d**: February 2016). Bilateral lung infiltrates with ground-glass attenuation (**a**). Bilateral infiltrates and dense consolidations aggravated (**b**). Minimal absorption compared to day 14 (**c**). Dense consolidations were significantly absorbed (**d**)

The patient had a fever of 38 °C and sought medical care at a local clinic with complaints of fever, chills, wheezing, and a productive cough. No significant findings were noted on chest X-ray (Fig. 1), and the patient was offered symptomatic treatment and discharged. He returned to the local clinic one week later because of worsening symptoms. Arterial blood gas analysis showed type I respiratory failure. A thoracic computed tomography (CT) scan reported bilateral lung infiltrates with ground-glass attenuation (Fig. 2a). The temperature climbed to 40 °C and the patient was then transferred to the respiratory intensive care unit (RICU).

After transfer to the RICU (day 1), the patient received non-invasive positive pressure ventilation (NIPPV) for respiratory support with continuous positive airway pressure at 4 cmH_2_O (FiO_2_ 50 %), and high-flow nasal cannula oxygen supplement (FiO_2_ 50 %) between gaps. Chest auscultation demonstrated bibasilar crepitation. An arterial blood sample was acquired under NIPPV support (FiO_2_ 50 %), and blood gas analysis showed the following: pH, 7.39; PaCO_2_, 36 mmHg; PaO_2_, 88 mmHg; and A-a O_2_ gradient, 68 mmHg. The leukocyte count was 12.2 × 10^9^/L. The procalcitonin level was 22.09 ng/ml. Other laboratory findings included the following: serum urea nitrogen, 30.83 mmol/L; serum creatinine, 478.50 umol/L; potassium, 5.3 mmol/L; and lactic acid dehydrogenase, 729 U/L. The serum 1,3-β-D-glucan level was > 1000 pg/ml (beyond the testing range). The CD4^+^ T-cell count was 46 cells/mm^3^. HIV was excluded by real-time polymerase chain reaction (PCR) analysis. *Pneumocystis jirovecii* was visualized under light microscopy in both induced sputum and bronchoalveolar lavage fluid (BALF) with Gomori methenamine silver staining. Cytomegalovirus (CMV)-pp65 antigen and CMV-DNA were positive in blood samples. Induced sputum and BALF were collected for real-time PCR analysis, yielding positive *P. jirovecii* DNA and CMV-DNA. A high-resolution CT (HRCT) scan on day 14 demonstrated aggravation of the bilateral lower lobe consolidation (Fig. 2b).

Because of the low oxygen index (PaO_2_/FiO_2_ = 166 mmHg) and infiltrates, the patient was thought to have developed moderate acute respiratory distress syndrome (ARDS), and a 21-day adjunctive corticosteroid therapy was initiated on day 1. Methylprednisolone (80 mg IVggt qd) was administered for the first 5 days, then tapered to 40 mg IVggt qd for another 5 days, and 20 mg po qd for 11 days more. Because the patient was allergic to TMP-SMZ which is a first-line choice for PCP, a TMP-SMZ desensitization protocol (0.12 g po q6h for 2 days, 0.48 g q6h for 2 days, and 0.96 g q6h for 2 days) was instituted on day 2. Ulcers and hemorrhages were observed on the left side of the tongue on day 7, which was believed to be an adverse reaction to TMP-SMZ. Therefore, TMP-SMZ therapy was abandoned on day 7, and was subsequently replaced by a 21-day combination therapy of caspofungin (50 mg IVggt qd) and clindamycin (0.3 g IVggt q6h) from days 8 to 28. Ganciclovir was added to cover CMV infection. Other pathogens, such as bacteria, could not be excluded in this case, thus cefoperazone-sulbactam and moxifloxacin were added empirically.

**Fig. 3:**
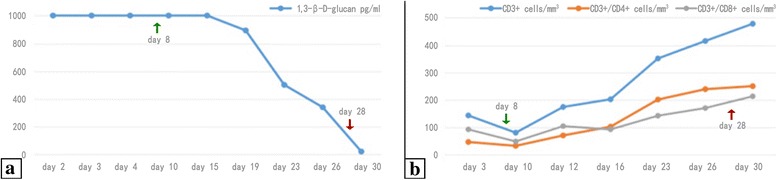
Serial monitoring of 1,3-β-D-glucan levels and absolute cell counts of T-cell subsets. The CD4^+^ T-cell count decreased when TMP-SMZ was discontinued, but gradually became normal (**b**), which was accompanied by a decreasing 1,3-β-D-glucan level (**a**). TMP-SMZ was stopped on day 7. Green arrows indicate the initiation of caspofungin and clindamycin on day 8. Red arrows indicate the cessation of the antifungal treatment on day 28

The patient’s condition gradually remitted and the oxygen index improved. The patient was transferred back to the general ward on day 33. The 1,3-β-D-glucan levels and absolute cell counts of T-cell subsets were carefully monitored. The CD4^+^ T-cell count decreased when TMP-SMZ was discontinued, but gradually normalized, which was accompanied by a decreasing 1,3-β-D-glucan level (Fig. 3). *Pneumocystis jirovecii* was undetectable microscopically in induced sputum on day 9. The PCR became negative for sputum *P. jirovecii* and CMV-DNA on days 13 and 29, respectively. On day 20, HRCT revealed that the upper lobe infiltrates and dense consolidations in the lower lobes were absorbed compared to the last scan (Fig. 2c). A follow-up HRCT was arranged on day 90, which showed significant absorption of the dense consolidations (Fig. 2d). The symptoms were nearly relieved by the time he was discharged, with the exception of occasional coughing. Because the patient restored his normal CD4^+^ T-cell count, secondary prophylaxis for PCP was not needed.

## Discussion

*Pneumocystis jirovecii* (formerly *Pneumocystis carinii*) was initially classified as a protozoan parasite, and molecular and genetic evidence categorized *P. jirovecii* among the fungi. Although *Pneumocystis* cannot be cultivated on standard artificial media and the lifecycle of the organism is unclear, *Pneumocystis* does share some biological characteristics with protozoa. Indeed, two apparently distinctive forms can be observed under microscope (trophic and cystic forms) [8]. Although trophic forms are predominant in infected tissues, glucans are only found in cystic forms [9].

Clinical manifestations, and laboratory and imaging studies are not pathognomonic in PCP, thus a heightened clinical suspicion should be maintained in patients known to be HIV-infected and immunosuppressed. HRCT may be valuable in assessing lung injury and the severity of PCP [10, 11]. A definitive diagnosis of PCP requires the detection of trophic and/or cystic forms of *P. jirovecii* at direct microscopic examination of lower respiratory tract samples, such as induced sputum, BALF and lung biopsy specimens [4]. With the development of PCR technology, especially real-time PCR assays, the detection of *P. jirovecii* DNA in respiratory samples has become an essential part of the laboratory diagnosis of PCP [4, 12]. Nevertheless, due to its high sensitivity, PCR may allow detection of *P. jirovecii* in latent infections without PCP, which decreases its specificity [13].

All polysaccharides of the fungal cell wall are different from those produced by mammalian cells. Among them, 1,3-β-D-glucan is a reliable adjunctive diagnostic marker for PCP, but not a prognosis predictor [14]; negative results are useful to rule out PCP in HIV patients [15]. Nevertheless, Kamada et al. [16] reported an HIV-infected patient with PCP and normal 1,3-β-D-glucan levels throughout the course of infection, which might reflect an early phase infection with limited lung injury. Interpreting 1,3-β-D-glucan results among non-HIV individuals should be done with care and in parallel with other clinical findings [15]. Furthermore, 1,3-β-D-glucan could be elevated in other invasive fungal diseases and may obscure the diagnosis.

In HIV-infected patients, PCP rarely occurs when the CD4^+^ T-cell count is > 200 cells/mm^3^ [17]. Moreover, CD4^+^ T-cell counts are of concern when initiating and discontinuing PCP prophylaxis in HIV-infected individuals [4]. Yet, consensus about CD4^+^ T-cell counts has not been well-established towards PCP unrelated to HIV. Nevertheless, non-HIV patients with CD4^+^ T-cell counts < 200 cells/mm^3^ appear to be at increased risk for developing PCP [18], thus it is reasonable to monitor CD4^+^ T-cell counts in such patients.

TMP-SMZ, an antibiotic used to treat a variety of infections, is the first-line drug for PCP prophylaxis and treatment, but treatment failure may occur because of dihydropteroate synthase and dihydrofolate reductase mutations during the course of the treatment [19, 20]. Adverse effects of TMP-SMZ are also common, such as fever, rash, nausea, vomiting, transaminase elevation, and more serious toxicities, including neutropenia, thrombocytopenia, Stevens-Johnson syndrome, and toxic epidermal necrolysis [21]. With mild adverse reactions, TMP-SMZ should be continued with a gradual dose increment (desensitization) or at a reduced dose or frequency. TMP-SMZ therapy should be aborted in patients with possible or definite life-threatening reactions [4].

Echinocandins are antifungal agents that non-competitively inhibit 1,3-β-D-glucan synthase [22]. Thus, echinocandins are toxic to fungi in which the glucans play an important role in maintaining the integrity of the fungal cell wall [22] and partly contribute to the host inflammatory response in the lung [23]. Caspofungin used alone or with low-dose TMP-SMZ has been shown to be efficient in treating PCP on experimental animal PCP models [5, 6]. Unlike TMP-SMZ, which primarily eliminates trophic forms of *Pneumocystis*, caspofungin clears cystic forms which might play a key role in transmission [7]. Nevertheless, the clinical use of echinocandins, such as caspofungin, against *P. jirovecii* infection is still controversial. Utili et al. [24] reported 4 cases of solid organ transplant recipients infected by PCP who were treated by caspofungin and TMP-SMZ with favorable outcomes. While Utili et al. [24] summarized 8 reported cases involving echinocandin-containing regimens for PCP before 2007, two lymphoblastic leukemia patients died in spite of prolonged echinocandin treatment in addition to other anti-*Pneumocystis* therapies. In a retrospective analysis among 80 HIV-PCP patients, 10 of whom had confirmed PCP microbiologically, received caspofungin-based salvage therapies, and showed satisfactory outcomes, one patient died with bilateral pneumothoraces [25].

Clindamycin combined with primaquine has activity against *P. jirovecii*, although the mechanism is still unclear. In protozoa, clindamycin targets protein synthesis in a parasite-specific organelle (the apicoplast) [26], which is related to mitochondrial function and the lifecycle of the organism. In addition, reduction of protein and nucleic acid synthesis has been observed in *Plasmodium falciparum* when exposed to clindamycin [27]. Primaquine could interfere with the microbial electron transport system by generating quinone metabolites and superoxides in vivo [28], which may prevent the proliferation of *P. jirovecii.* A previous study by Queener et al. [29] demonstrated a higher efficacy of clindamycin combined with primaquine compared to the use of each drug alone for treatment and prophylaxis of PCP in rat models. Clindamycin/primaquine regimens, the clinical efficacy of which have been proven by clinical trial [30], appear to have the highest efficacy among alternative therapies for PCP [31] and are now used as second-line therapy in PCP management [4].

A moderate-to-severe PCP infection is defined as a PaO_2_ < 70 mmHg at room air or an A-a O_2_ gradient ≥ 35 mmHg [4]. Because our patient’s blood gas analysis was tested under NIPPV, the severity of PCP had to be evaluated with all of the clinical findings. In the current case, this middle-aged man, who had undergone prolonged corticosteroid therapy for an underlying IgA nephropathy, was diagnosed with PCP and developed ARDS. The adjunctive corticosteroid dose needed to be individualized to balance anti-inflammatory against immunosuppressed effects. Thus, we began high-dose methylprednisolone tapering. The patient failed desensitization to TMP-SMZ. Given that primaquine is not routinely available in our hospital, alternative therapy with caspofungin plus clindamycin was initiated. The only report of concomitant use of caspofungin with clindamycin for PCP involves a patient who did not respond to combination therapy and the infection was eventually controlled following TMP-SMZ desensitization [32]. Our patient showed an impressive response to the treatment and ultimately recovered.

Because the microscopic images of respiratory tract specimens were not attached to the patient’s documents, we have no way to determine how the trophic and cystic forms changed during the course of treatment, but elevated 1,3-β-D-glucan levels implied that cysts had been producing glucans on which caspofungin might have an effect. The efficacy of clindamycin could not be determined clearly in our case. Given that clindamycin improves the outcome together with primaquine, the efficacy with caspofungin may warrant further investigation. Our patient showed a concomitant CMV infection and received ganciclovir for the treatment, while clinical study suggested that concomitant CMV infection in non-HIV related PCP did not affect prognosis and antiviral drugs might be unnecessary [33].

## Conclusion

In summary, this new combination therapy of caspofungin plus clindamycin in managing PCP may be considered in patients who fail standard treatment. Monitoring 1,3-β-D-glucan is of more convenience than other forms of testing, such as BALF, and may provide early diagnostic clues. Based on our case and a review of the literature, we suggest that highly elevated 1,3-β-D-glucan levels may be a predictor of a satisfactory caspofungin response to PCP.

## Abbreviations

ARDS: Acute respiratory distress syndrome
BALF: Bronchoalveolar lavage fluid
CMV: Cytomegalovirus
CT: Computed tomography
HRCT: High-resolution computed tomography
NIPPV: Non-invasive positive pressure ventilation
PCP: *Pneumocystis* pneumonia
PCR: Polymerase chain reaction
RICU: Respiratory intensive care unit
TMP-SMZ: Trimethoprim-sulfamethoxazole
